# Abundance and Dynamics of Antibiotic Resistance Genes and Integrons in Lake Sediment Microcosms

**DOI:** 10.1371/journal.pone.0108151

**Published:** 2014-09-23

**Authors:** Björn Berglund, Ghazanfar Ali Khan, Richard Lindberg, Jerker Fick, Per-Eric Lindgren

**Affiliations:** 1 Department of Clinical and Experimental Medicine, Linköping University, Linköping, Sweden; 2 Department of Chemistry, Umeå University, Umeå, Sweden; 3 Department of Microbiology, Medical Services, County Hospital Ryhov, Jönköping, Sweden; Oak Ridge National Laboratory, United States of America

## Abstract

Antibiotic resistance in bacteria causing disease is an ever growing threat to the world. Recently, environmental bacteria have become established as important both as sources of antibiotic resistance genes and in disseminating resistance genes. Low levels of antibiotics and other pharmaceuticals are regularly released into water environments via wastewater, and the concern is that such environmental contamination may serve to create hotspots for antibiotic resistance gene selection and dissemination. In this study, microcosms were created from water and sediments gathered from a lake in Sweden only lightly affected by human activities. The microcosms were exposed to a mixture of antibiotics of varying environmentally relevant concentrations (i.e., concentrations commonly encountered in wastewaters) in order to investigate the effect of low levels of antibiotics on antibiotic resistance gene abundances and dynamics in a previously uncontaminated environment. Antibiotic concentrations were measured using liquid chromatography-tandem mass spectrometry. Abundances of seven antibiotic resistance genes and the class 1 integron integrase gene, *intI1*, were quantified using real-time PCR. Resistance genes *sulI* and *ermB* were quantified in the microcosm sediments with mean abundances 5 and 15 gene copies/10^6^ 16S rRNA gene copies, respectively. Class 1 integrons were determined in the sediments with a mean concentration of 3.8×10^4^ copies/10^6^ 16S rRNA gene copies. The antibiotic treatment had no observable effect on antibiotic resistance gene or integron abundances.

## Introduction

When antibiotics were introduced for therapeutic use in the 1930s, their success at treating bacterial infections completely changed clinical practice and saved countless lives. However, the use of antibiotics also drives the development and enrichment of antibiotic resistance determinants. Today, antibiotic resistant bacteria are widespread and the rapid dissemination of multi-drug resistance in particular, has brought considerable concerns as to whether humanity is about to return to the pre-antibiotic era [1].

While studies investigating antibiotic resistance have classically been constrained to clinical contexts, it has recently been recognised that the environment is likely to play a major role in the development and dissemination of antibiotic resistance genes (ARGs). Antibiotics and ARGs originate from environmental bacteria, and it is likely that anthropogenic contamination with antibiotics can drive selection for resistance, which in turn may find its way into pathogenic bacteria [2]. Water environments in particular are important in the circulation and accumulation of antibiotic discharge and are therefore likely to serve as hotspots for development and dissemination of ARGs [3]. ARGs in water environments have been reported to correlate with anthropogenic activities [4]–[6], and extremely high concentrations of antibiotic pollutants have been shown to correlate to high ARG levels [7]–[8]. Furthermore, it has been shown that antibiotic concentrations below minimum inhibitory concentrations (MICs) can select for resistant bacteria [9]. It is clear that studies are needed to elucidate what effect low-level antibiotic contamination (i.e. concentrations ranging from ng/L to low µg/L) has on the resistance development in the environmental bacterial community.

Mobile genetic elements containing resistance determinants are also considered important for widespread dissemination of ARGs [2]. Integrons are a type of genetic assembly platform often associated with mobile genetic elements. They often harbour ARG cassettes and have the ability to capture new gene cassettes with enzymes known as integrases [10]. Prevalence of integrons has been reported to be elevated in anthropogenically impacted sites, indicating a potential correlation between pollution and the ability of antibiotic resistance to be disseminated [11]–[13]. The total number and function of different environmental gene cassettes available for integrons are still largely unknown. Previous studies have estimated the total number of different genes cassettes to be over 2,000 in 50 m^2^ of soil [14] and about 1,000 in marine sediments [15]. Even though many integron gene cassettes are likely to encode non-resistance functions, the huge diversity of cassettes which environmental integrons are likely to be able to access suggests that these genetic elements play a significant role in bacterial adaptability [14].

Microcosms are useful tools in ecological studies involving large and complex microbial systems. The main advantages of microcosms are their high capacity for experimental control and that they enable the study of selective processes in highly complex systems [16]. The effects of antibiotics on a bacterial community and the dissemination of resistance in aquatic environments are typical phenomena which can be studied with microcosms. Related examples include studies which have investigated tetracycline resistance in agricultural soils [17], effects of amoxicillin on bacteria in manured soil [18] and dissemination of a multi-drug resistance plasmid in wastewater sludge [19].

The aim of this study was to investigate the effects of low, environmentally relevant levels of antibiotics (i.e. ng/L to low µg/L) on the abundance of ARGs and class 1 integrons of a bacterial community from a water environment only lightly affected by human activities. Microcosms consisting of water and sediment from Lake Nydala, Sweden, were set up under standardised laboratory conditions. Mixtures of antibiotics of different concentration levels were added to the microcosms, and samples were taken over 100 days. Antibiotic concentrations were determined in the water phase and in the sediment phase using liquid chromatography-mass spectrometry (LC-MS). Seven different ARGs and *intI1*, the integrase gene on class 1 integrons, were determined in the sediment phase using quantitative real-time PCR.

## Materials and Methods

### Microcosms

Sediment and water were taken from Lake Nydala (63° 49.081N, 20° 20.784E) during ice cover in January 2009. No specific permission was required for the sampling and no endangered or protected species were involved. Lake Nydala does not receive any sewage water discharges. Sediment was taken with a HON-Kajak corer [20] and given that the sampling depth was restricted to 5 cm, the sediment taken was mainly under oxic conditions. The sediment was wet-sieved (4 mm mesh), homogenised and subsamples were taken for measuring dry weight (105° C) and loss on ignition. The experiments were performed similar to OECD Guideline 308 [21] and consisted of 500 mL amber glass flasks (Schott, Mainz, Germany) filled with 200 g of sediment and 300 mL of lake water. Microcosms were equilibrated for seven days prior to spiking with antibiotics. During the equilibration and the study period, the microcosms were slowly shaken and kept at 10° C. At the beginning of the study period, antibiotics were spiked into the water phase in each microcosm, at the nominal concentrations shown in Table 1. The antibiotics used were azithromycin (AZI), ciprofloxacin (CIP), clarithromycin (CLA), clindamycin (CLI), doxycycline (DOX), erythromycin (ERY), norfloxacin (NOR), oxytetracycline (OXY), sulfamethoxazole (SUL), tetracycline (TET) and trimethoprim (TRI), all of which were purchased from Sigma-Aldrich (Steinheim, Germany) and of HPLC grade purity (>98%). Samples were taken of water and sediments immediately prior to spiking, and after 1, 7, 14, 28, 50, 76 and 100 days.

**Table 1:** The experimental set-up consisted of three series of microcosms with varying concentrations of antibiotics added (1x, 10x and 1000x), and additional control microcosms without antibiotic addition.

Antibiotic	Nominal concentration (µg/L)		
	1x	10x	1000x
Azithromycin (AZI)	0.01	0.1	10
Ciprofloxacin (CIP)	0.02	0.2	20
Clarithromycin (CLA)	0.04	0.4	40
Clindamycin (CLI)	0.02	0.2	20
Doxycycline (DOX)	0.01	0.1	10
Erythromycin (ERY)	0.2	2	200
Norfloxacin (NOR)	0.1	1	100
Oxytetracycline (OXY)	0.04	0.4	40
Sulfamethoxazole (SUL)	0.1	1	100
Tetracycline (TET)	0.1	1	100
Trimethoprim (TRI)	0.1	1	100

### Antibiotic quantification using LC-MS/MS

Water samples were pre-treated as described in [22]. In short, the samples were filtered through a 0.45 µm membrane filter, acidified and isotopically labelled internal standards were added to each sample prior to solid phase extraction. Before extraction of the sediment samples (0.1 g, dry weight) 50 ng of each internal and surrogate standard were added. Extraction was sequentially performed first using 1.5 mL ethylacetate and methanol (1∶1 mixture) followed by 1.5 mL methanol and water (7∶3 mixture) with 5% triethylamine. Samples were homogenised for four minutes at 42,000 oscillations per minute, using a Mini Beadbeater (Biospec, Bartlesville, OK) with zirconium beads and then centrifuged at 14,000 revolutions per minute for 10 min. The protocol was followed for both eluent mixtures, the supernatants were combined, evaporated to 20 µL and reconstituted in 1 mL water and acetonitrile (95∶5 mixture) with 0.1% formic acid.

Antibiotic concentrations in the water and sediment samples were determined by chemical analysis using liquid chromatography-tandem mass spectrometry, as described in [22]. In short, a triple stage quadrupole MS/MS TSQ Quantum Ultra EMR (Thermo Fisher Scientific, San Jose, CA) coupled with an Accela LC pump (Thermo Fisher Scientific) and a PAL HTC autosampler (CTC Analytics AG, Zwingen, Switzerland) were used as analytical system.

### DNA extraction

200 mg of sediment from each sample was weighed and DNA was extracted using FastDNA SPIN Kit for Soil and the FastPrep Instrument (MP Biomedicals, Santa Ana, CA) according to the manufacturer's protocol. Extracted DNA was stored in −20°C before subsequent analyses. DNA concentrations in extracted DNA from different amounts of starting sediment were measured using Quant-iT PicoGreen dsDNA reagent (Invitrogen, Carlsbad, CA) to ensure a linear yield of extracted DNA.

### Gene quantification with real-time PCR

Quantitative real-time PCR was used for determining gene concentrations in the extracted DNA from the microcosm sediments. The analysed genes were tetracycline resistance genes *tetA* and *tetB*, macrolide-lincosamide-streptogramin B resistance gene *ermB*, sulphonamide resistance gene *sulI*, trimethoprim resistance gene *dfrA1*, quinolone resistance gene *qnrS*, glycopeptide resistance gene *vanB* and the integrase gene on class 1 integrons, *intI1*. Gene concentrations were normalised by the measured 16S rRNA gene concentration of each sample. 16S rRNA gene concentrations were normalised against extraction mass when used separately in statistical analysis. All real-time PCRs were carried out on a CFX96 Real-Time PCR Detection System (Bio-Rad Laboratories, Hercules, CA). Quantification method, primers, primer concentrations and thermal cycling protocols were used as in [22].

### Statistical methods

Two-way Repeated Measures ANOVA was used to determine the effects of initial antibiotic level and time spent in the microcosms on gene concentrations. Where time was found as a significant factor, One-way ANOVA with subsequent Bonferroni multiple comparisons test was used to determine the relevant differences. All statistical analyses were carried out using Prism 5 for Windows v.5.00.

### Ethics statement

No specific permits were required for the described study. The field study did not involve any endangered or protected species.

## Results

### Quantification of antibiotics

**Figure 1:**
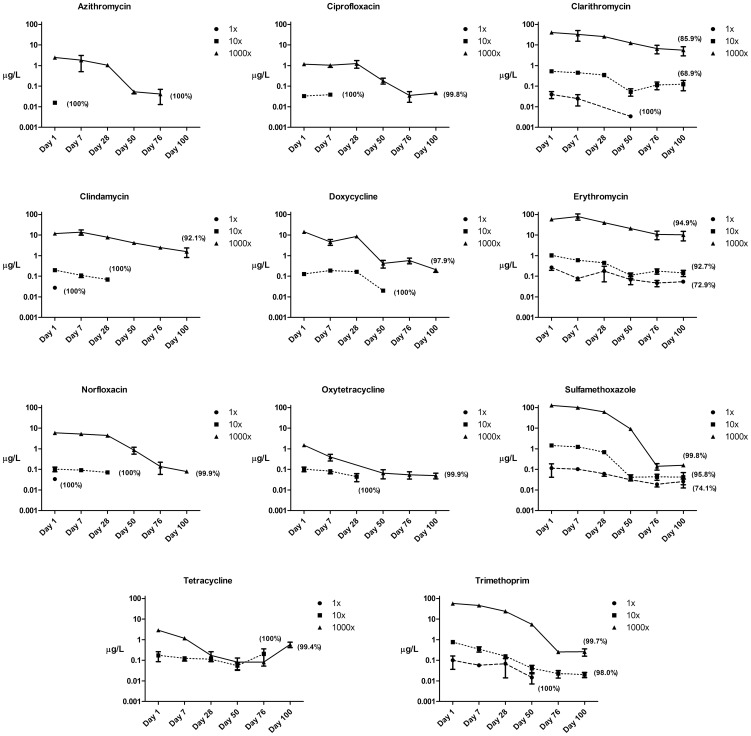
Antibiotics quantified in the water phase over 100 days in microcosms for three microcosms (1x, 10x and 1000x antibiotic concentration). Error bars indicate the standard error of the mean. The reduction (relative decrease of the initial concentration as compared to day ‘100’) is presented in parenthesis after each line. Sampling points without dots indicate that the antibiotic concentration was below the limit of quantification except for the quantification of oxytetracycline at day ‘28’ in the 1000x microcosm which could not be performed due to sample loss.

The antibiotic concentrations in the water phase declined over time in all microcosms (Fig. 1). For the majority of antibiotics, the reduction in concentration from the initial added concentration was close to 100% at day ‘100’. Only CLA in the 10x and 1000x microcosms (68.9% and 85.9% reduction respectively) and ERY and SUL in the 1x microcosm (72.9% and 74.1% respectively) showed antibiotic reductions below 90%. The ratios of the antibiotic concentrations in the microcosms of different concentrations adhered fairly well to the theoretical values. The means±SEM of the ratios (based on all sampling points where quantification was possible) were 6.4±1.3, 550±103 and 69±8.9 for the 10x∶1x, 1000x∶1x and 1000x∶10x microcosms respectively.

In the sediment phase, no antibiotics were detected in the microcosms with 1x concentration (Table 2). In the microcosms with 10x concentration, only CLA, CLI and TRI could be detected, and only at day ‘100’. In the 1000x microcosms, CLA, CLI and TRI were detected at all days sampled after antibiotic addition (means 13, 6.8 and 6.9 µg/g respectively). SUL was also detected, but only in days ‘1’ and ‘7’. AZI, CIP, DOX, ERY, NOR, OXY and TET were not detected at any sampling point in the sediments.

**Table 2:** Antibiotics quantified in the sediment phase over 100 days in microcosms of differing initial antibiotic concentrations (1x, 10x and 1000x).

				Concentration by day (ng/g)			
		1	7	28	50	76	100
	AZI	-	-	-	-	-	-
	CIP	-	-	-	-	-	-
	CLA	-	-	-	-	-	-
	CLI	-	-	-	-	-	-
	DOX	-	-	-	-	-	-
1x	ERY	-	-	-	-	-	-
	NOR	-	-	-	-	-	-
	OXY	-	-	-	-	-	-
	SUL	-	-	-	-	-	-
	TET	-	-	-	-	-	-
	TRI	-	-	-	-	-	-
							
	AZI	-	-	-	-	-	-
	CIP	-	-	-	-	-	-
	CLA	-	-	-	-	-	2099
	CLI	-	-	-	-	-	254
	DOX	-	-	-	-	-	-
10x	ERY	-	-	-	-	-	-
	NOR	-	-	-	-	-	-
	OXY	-	-	-	-	-	-
	SUL	-	-	-	-	-	-
	TET	-	-	-	-	-	-
	TRI	-	-	-	-	-	1647
							
	AZI	-	-	-	-	N.A.	-
	CIP	-	-	-	-	N.A.	-
	CLA	3161	14454	6937	8376	N.A.	30373
	CLI	1972	7660	3725	8698	N.A.	12013
	DOX	-	-	-	-	N.A.	-
1000x	ERY	-	-	-	-	N.A.	-
	NOR	-	-	-	-	N.A.	-
	OXY	-	-	-	-	N.A.	-
	SUL	1606	1432	-	-	N.A.	-
	TET	-	-	-	-	N.A.	-
	TRI	3429	17374	5113	3191	N.A.	5286

‘-’: below limit of quantification.

‘N.A.’: not analysed due to sample loss.

No antibiotics were detected in the day ‘0’ samples (i.e. before antibiotics were added) or in the controls (i.e. the microcosms in which no antibiotics were added) in either the water or the sediment phase (data not shown).

### Gene quantification with real-time PCR

Sulphonamide resistance gene *sulI* was detected in 16 out of 75 samples (Table 3). Concentrations were sufficiently high for quantification in only 6 out of these 16 samples (mean value: 5 gene copies/10^6^ 16S rRNA gene copies). Macrolide-lincosamide-streptogramin B resistance gene *ermB* was detected in 4 out of 75 samples (Table 3), with sufficiently high concentrations to be quantifiable in 3 of these samples (mean value: 15 gene copies/10^6^ 16S rRNA gene copies). No trends in prevalence or concentration could be observed for either *sulI* or *ermB* in relation to time points or in relation to antibiotic concentration. Trimethoprim resistance gene *dfrA1* could only be detected in 1 sample (at day 1 in a microcosm treated with antibiotics at the 1000x concentration) at a concentration below the limit of quantification (data not shown). Tetracycline resistance genes *tetA* and *tetB*, glycopeptide resistance gene *vanB* and quinolone resistance gene *qnrS* could not be detected in any sample.

**Table 3:** Quantification and detection of antibiotic resistance genes *sulI* and *ermB* in microcosm sediments with values in units of gene copies/10^6^ 16S rRNA gene copies.

		Day 1	Day 7	Day 28	Day 50	Day 76	Day 100
	0x	3	+	+	-	+	4
*sulI*	1x	+	-	7	-	4	+
	10x	+	-	-	-	-	+
	1000x	-	-	+	-	5	-
	0x	-	-	11	-	-	-
*ermB*	1x	-	-	+	6	27	-
	10x	-	-	-	-	-	-
	1000x	-	-	-	-	-	-

‘-’: below limit of detection.

‘+’: detected, but below quantification limit.

**Figure 2:**
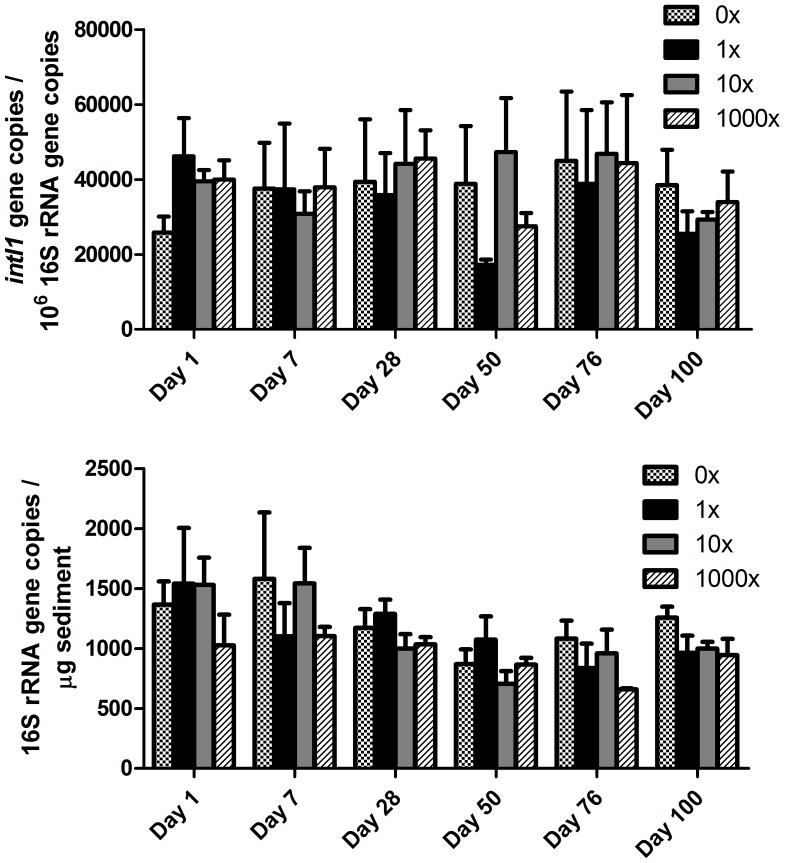
Mean by day of *intI1* and 16S rRNA gene concentrations measured in the microcosm sediments. Different coloured bars denote the microcosm which had different starting concentrations of antibiotics added. Error bars denote the standard error of the mean.

*intI1*, the integrase gene on class 1 integrons, was quantified in all samples (mean value: 3.8×10^4^ gene copies/10^6^ 16S rRNA gene copies) (Fig. 2). Concentrations of *intI1* did not differ significantly between different time points or between different antibiotic concentration levels. The mean concentration of *intI1* in the day ‘0’ samples (n = 3), i.e. before antibiotics were added, was 4.9×10^4^ gene copies/10^6^ 16S rRNA gene copies (data not shown).

Concentrations of 16S rRNA gene copies did not appear to vary with different initial concentrations of input antibiotics, although variation could be significantly attributed to the number of days in the microcosms (p<0.001). 16S rRNA gene copy concentrations were lower at day ‘50’ and day ‘76’ than at day ‘1’ (p<0.05) (Fig. 2).

## Discussion

Initial antibiotic concentrations in the gathered water and sediments were below the limit of quantification. Furthermore, no antibiotics were detectable in the control microcosms during the run of the experiment. In the water phase, the ratio of antibiotic concentrations between the microcosms of different starting concentrations also tended to correspond to the initial ratio. The antibiotic quantification also showed that concentrations were reduced over time (in most microcosms, and for most antibiotics, by almost 100% over 100 days) in the water phase. The same trend was not observable for the antibiotics which were quantifiable in the sediment phase. Instead, antibiotic concentrations tended to either increase (i.e. accumulate) or remain unchanged, likely due to saturation of the sediments. Due to the high limit of quantification in the sediments, it is difficult to speculate how much of the antibiotics remained in the sediment. It is unclear how antibiotics bound to sediments affect bacteria, although it has been reported that even tightly adsorbed to clay particles, antibiotic compounds are biologically active [23].

ARGs could only be quantified and detected in a few of the samples. *sulI* was the most prevalent gene (detected in 16 out of 75 samples) and the mean of the concentration out of the quantifiable concentrations (n = 6) was 5 gene copies/10^6^ 16S rRNA gene copies. This is comparable to levels measured in [4] where *sulI* was measured in river sediments from a pristine location at concentrations around 10^0^ gene copies/10^6^ rRNA gene copies. *ermB* was also detected in a few samples and *dfrA1* in only one sample. *tetA*, *tetB*, *vanB* and *qnrS* were not detected in any sample. It should be noted that *tetA* and *tetB* have been found to occur stably over time in Swedish wastewaters [24], and *vanB* have been found in Swedish hospitals [25], although these environments can hardly be described as pristine. That these genes are not present in sediments collected from an environment only tangentially perturbed by anthropogenic activities is not surprising, although it should be noted that ARGs have been shown to exist in pristine environments [26]–[27]. The most prevalent ARG found, *sulI*, did not appear to increase in abundance over time, indicating that the antibiotics had no effect.

The class 1 integron integrase gene *intI1* was quantified in all samples. Concentrations did not significantly vary either when looking at different antibiotic concentrations or over time. This indicates that the antibiotics did not affect the concentration of *intI1*. This is noteworthy since several earlier studies have shown that integron abundances increase in anthropogenically impacted environments [6], [11], [13]. Notably, the mean concentration in the day ‘0’ samples was in the same range as the mean for the concentrations of later dates (i.e. after antibiotics were added). This indicates that the abundance of the *intI1* gene was not particularly affected during the run-time of the experiment. Care should be taken when making this comparison however, since only three samples were used to calculate the day ‘0’ mean.

Time was determined to be a statistically relevant parameter affecting the concentration of the 16S rRNA gene in the microcosms (p<0.001). The trend was towards lower concentrations at the later days (i.e. day ‘50’ and day ‘76’) than day ‘1’, at the start of the experiment (p<0.05). The statistical tests applied indicated that this decline was independent of antibiotic treatment. It is likely that the declining concentration of 16S rRNA genes is indicative of a reduction in bacteria due to low nutritional content in the sediments, low oxygen availability or other environmental factors. In an environment where the bacterial community is dying due to a lack of vital nutrients, maintaining antibiotic resistance functionality may be too costly to be beneficial. In such a case, the decline in 16S rRNA genes may explain the observation that the ARG concentrations remain the same throughout the experiment. Another explanation could be that the composition of the bacterial community in the microcosms changed as bacteria with a lower 16S rRNA gene copy number became dominant at the expense of bacteria with a higher copy number. Although, even if this was the case, antibiotics could still be ruled out as having a significant effect on this development, as the same trends were seen in the control microcosms and the spiked microcosms, irrespective of antibiotic concentrations. If the decline in 16S rRNA gene copies is an indication that the bacteria in the microcosms did not grow, it may also explain why the antibiotics did not have a significant effect, as some antibiotics exhibit significantly less killing power on slow and non-growing bacteria [28].

The initial concentrations of antibiotics (defined as 1x concentration) added to the microcosms were chosen to mimic the concentrations commonly encountered in effluent-dominated surface waters and similarly perturbed aquatic environments. An increase in antibiotic resistance gene or integron abundance could not be observed in any of the concentration ranges. In [22] it was shown that antibiotic concentrations equal to the 10x level, did not affect resistance gene abundances in a series of constructed wetlands. In [9] it was shown that antibiotic resistant bacteria could be selected for at antibiotic concentrations below MIC-levels. This was performed for concentrations of TET at 15 µg/L and for CIP as low as 0.1 µg/L. These are concentrations lower by about one order of magnitude compared to the initial concentrations used in the 1000x microcosms. The lack of effect seen in this experiment at these concentrations could be explained by the possible lack of bacterial growth and that the bacterial community used in this experiment was considerably more complex.

The experiment parameters in this study were set to simulate a bacterial community in a previously unperturbed environment exposed to a mixture of antibiotics of concentrations likely to occur in an anthropogenic contamination event. The addition of antibiotics had no discernible effect on the abundance of resistance genes or integrons measured in this experiment (including the 16S rRNA gene) at any of the concentrations. That no effect could be seen in the microcosm which was exposed to a thousand times the environmentally relevant concentrations is notable, as the levels have been shown to select for resistant bacteria in less complex systems. Further studies are needed to establish what, if any, effect these higher antibiotic levels have on bacterial communities in pristine and lightly affected environments.
